# Data–driven analysis of Romania's renewable energy landscape and investment uncertainties

**DOI:** 10.1016/j.heliyon.2024.e27334

**Published:** 2024-03-07

**Authors:** Mihai Ciobotea, Ecaterina-Milica Dobrotă, Marian Stan, Delia Bălăcian, Silvius Stanciu, Adriana Dima

**Affiliations:** a Bucharest University of Economic Studies, Romania; b “Dunarea de Jos” University of Galați, Romania

**Keywords:** Energy production, Monte Carlo simulation, Public procurement, Green energy, Renewable energy

## Abstract

In recent years, there has been a significant transformation in the energy sector in the European Union as a whole. The shift towards producing renewable energy has had a significant impact on the economic development of most countries, requiring substantial investments through public procurement. This study aims to analyse the evolution of the energy sector in Romania from an economic perspective by using a data-driven approach. The data used in this research is collected from publicly available sources and pertains to energy production and public acquisitions in Romania, carried out through the Electronic Public Procurement System. By using a mixed approach, combining documentary analysis, literature review, and predictive modelling, the study reveals a shift towards more sustainable energy options. There is a significant decrease in the production of thermal power and an increase in solar and wind power. The findings provide an overview and potential scenario of Romania's electricity production levels in 2023, shedding light on the relative uncertainties associated with such a transition. The findings also suggest a clear and growing commitment in Romania towards the adoption of alternative energy sources, as reflected in the trends of public procurement. These procurement trends offer a valuable perspective on policy-making, investment planning, and progress monitoring in Romania's energy transition. Despite the inherent uncertainties in such a transition, the study demonstrates Romania's potential in terms of diverse sources for electricity production as well as the role of public procurement in achieving energy transformation.

## 1 Introduction

Economic development is inextricably linked to energy consumption. Industrial activity, but also transport (whether maritime, air, or road), are causes of the increase in the degree of pollution. The adoption of alternative energy as well as nuclear energy will create a cleaner environment. Worldwide, 10% of electricity is obtained from nuclear power plants [1].

The transition from conventional energy sources to sustainable ones, such as wind, solar, biofuels, and geothermal, as well as other low-carbon footprint alternatives, is the main objective of energy transformations [2]. The decisive role in achieving this transition is held by the state, which, through its dual capacity as both a legislator and an investor, possesses the ability to reform the energy system. Governments can establish energy efficiency standards for buildings, transportation norms, and regulations to reduce emissions, and impose pollution limits on industrial activities. Investments made by public entities in photovoltaic systems, wind turbines, nuclear power plants, as well as other systems utilizing alternative energy sources, will foster the growth of the industry in this field, ultimately leading to the creation of new jobs. The current European context is fostering transformation within the energy sector, encompassing endeavours such as augmenting the portion of renewable energy sources and enhancing energy efficiency through investments in digitalization, along with the development of requisite infrastructure to underpin this transition. The REPowerEU plan underpins the current energy regulations and actions, setting ambitious targets for Member States in terms of diversifying energy supply, strengthening infrastructure, reducing energy consumption, and increasing energy efficiency, as well as creating new capacity for renewable energy and hydrogen from renewable sources. The main objective of the REPowerEU plan is to minimise Europe's dependence on fossil fuel imports from Russia by accelerating measures to ensure the transition to clean energy and build a more resilient European energy system [3].

The measures at the European level must be integrated by national regulators within each Member State and tailored to suit the particular national energy mix. These measures are designed to complement the targets already established in the 2021 'Ready for 55′ package of proposals by Ref. [4], as well as to build upon the Recovery and Resilience Mechanism (RRM) as an integral component of the temporary financial instrument NextGenerationEU, introduced by the European Commission [5].

Romania has developed the National Recovery and Resilience Plan (NRRP) to be able to access the funds allocated by the €29.2 billion RRM, but also to establish which are the priority areas for investment to overcome the economic effects of the health crisis, to recover economically, and to increase resilience capacity [6]. Romania's Recovery and Resilience Plan involves spending €855 million on both reforms and investments to promote clean energy production. This will involve phasing out the use of coal and lignite power production, as well as investing in renewable energy and hydrogen. Moreover, an additional €2.7 billion will be allocated towards making buildings more energy-efficient and ensuring that they can withstand seismic activity [7].

The investments planned to be made through the NRRP involve significant public purchases carried out by the majority of the contracting authorities, in order promote the switch towards the usage of renewable energy sources, the increase in energy efficiency of buildings, the reduction of fossil fuel consumption, with an effect on the reduction of pollution. In this sense, through the NRRP, significant amounts are allocated for sustainable transport (7620 million euros), including the purchase of electric/hydrogen buses and recharging stations for minibuses and electric buses.

This study seeks to address the following research questions.

RQ1 How has total electricity production, along with its components, evolved in Romania over the past few decades?

RQ2 What is the projected value of Romania's total energy production for 2023?

RQ3 To what extent, within the past five years, have public procurement contracts, as catalogued in the Electronic Public Procurement System (EPPS), for the procurement of alternative energy sources, aligned with Romania's commitment to the European Union's green energy objectives and its pursuit of carbon neutrality, as delineated in the National Recovery and Resilience Plan (NRRP)?

The paper undertakes a comprehensive analysis of public procurement activities within Romania's energy sector. The choice of the energy sector as the focus of this analysis is rooted in the wealth of publicly available data accessible through the Electronic Public Procurement System (EPPS). This reservoir of data facilitates a thorough exploration of executed contracts, providing a valuable resource for comprehending procurement dynamics. The primary objective of this research is to examine the patterns and trends characterizing procurement activities in the energy sector, with a particular emphasis on discerning the trajectory of 'green' investments. Through this approach, the study aims to illuminate Romania's commitment to realizing the Sustainable Development Goals outlined in the NRRP. These objectives revolve around the promotion of alternative electricity sources and the concomitant reduction of environmental pollution, rendering the energy sector a focal point of interest for the authors.

The paper analyses the transition within the Romanian energy sector by examining the evolution of energy production within the country. It employs this data to project future trends, utilizing the Monte Carlo simulation method for insight. According to Yang, Ji and Geng, Monte Carlo is considered the most widely used method to generate a very large number of scenarios in a short time [8]. Due to the large amount of data that can be processed, Monte Carlo simulation is used in various fields, including energy. In the US, Zhang, Garapati and Doughty applied this numerical simulation method to the study of geothermal exploration [9]. In Japan, Togashi built a model to predict the energy consumption of a building using the Monte Carlo method [10].

The study consists of an analysis of historical data related to energy production and relevant public acquisitions in Romania. Furthermore, the authors propose a simulation-based approach in order to assess the energy production level for 2023 and the dynamics of public acquisitions of renewable energy. In this regard, linear regression and Monte Carlo simulation techniques have been used.

Furthermore, an analysis of the purchases registered in the Electronic Public Procurement System (EPPS), which are subject to contract in this economic sector, is carried out. With more than a year having passed since the approval of the NRRP, it is interesting to see where Romania stands in relation to the plan of reforms, policies and investments set out in the energy chapter.

The analysis is useful for decision-makers, researchers and the general public interested in Romania's energy transformation. Also, the study is useful to any researcher, at least from Europe, considering that, like Romania, the other EU member states carry out public procurement according to the rules established by Directive no. 24/2014 [11], respectively Directive no. 25/2016 (in the case of sectoral purchases) [12]. The similarity of the public procurement process, at the EU level, allows the analysis in this paper to help understand the evolution of green procurement in other countries as well.

Given that any economic entity, irrespective of its location, can engage in procedures initiated through the EPPS platform, the study unveils the potential for commercial enterprises to establish contracts with public institutions in Romania. Annually, the state allocates significant sums for the carrying out of various public procurements necessary to fulfil the objectives for which the institution was established [13]. These contracts pertain to public procurement within the energy sector, encompassing items such as solar panels, electric buses and hybrids, as well as the utilization of geothermal water, among others.

The necessity of this research lies in addressing a gap in understanding the Romanian energy sector, examining it through the lenses of economic development and public procurement. The significance of the study consists in exploring the available options for sustainable energy at the national level. This is particularly relevant in the context of the EU strategy towards renewable energy and the challenges incurred by energy transformation.

The novelty of this research resides in the fact that it is a data-driven approach, using a combination of documentary analysis, literature review and predictive modelling, in particular through a Monte Carlo simulation. Such a methodical approach permits a detailed exploration of Romania's energy production trends along with that of dynamics of Romania's renewable energy public procurement, providing new insights on the analysis of the energy sector's evolution, especially in the context of Romanian commitment towards European Union green energy objectives.

A number of recent studies have explored Romania's renewable energy landscape and the uncertainties surrounding investment in the sector. For example, Cîrstea et al. [14] and Lupu et al. [15] highlighted the potential for further development, emphasizing the need for a support mechanism [14] and identifying key factors for solar energy growth [15]. Other authors argued that the sector's financial performance was challenging, with the latter attributing this to policy changes [16,17].

However, studies have not analysed the renewables sector from a public procurement perspective, and the current study fills this gap as well.

The main objectives of the study are to analyse the evolution of the Romanian energy sector and to project future trends, especially in the context of the role of public procurement in transitioning to renewable energy. The practical contribution lies in providing insights for policy-makers, investment specialists, researchers, and the public regarding Romania's energy transition.

The current research contributes to theoretical research on the transition to renewable energy and on the impact of public procurement. It also highlights the significance of sustainable energy sources in achieving environmental goals. The findings of this study, such as the projected value of Romania's total energy production for 2023 on different probability levels, have broader implications for understanding the impact of public procurement in achieving the transition to sustainable energy sources.

## 2 Literature review

Azam et al. argued that climate change issues force significant investments in clean energy and the elimination of those assets/facilities that produce high CO2 emissions [1]. The energy sector, although vital for economic development, stands as one of the major greenhouse gas producers, primarily due to its heavy reliance on fossil fuels. Lee et al. have stated that the identification and exploitation of renewable energy sources is a driver of technological innovations in this field [18].

Conventional systems for heating and ventilation of buildings, but also for the production of domestic hot water, are based on the use of electricity from the national electricity grid, but also on the consumption of natural gas and fossil fuels. These systems produce high carbon emissions, which have a negative impact on the environment.

Sustainable development objectives require the identification of alternative sources of energy, such as those from geothermal energy, photovoltaic systems, wind systems, biomass-pellet fuels, etc. The use of sustainable means of transport and clean heating systems, also represent objectives of sustainable development. Dyr, Misiurski and Ziolkowska found that the replacement of diesel buses for public transport with buses fuelled by compressed natural gas results in a significant reduction of greenhouse gases [19]. Brdulak, Chaberek and Jagodziński conducted an extensive analysis of public transport in the EU and determined that by 2050, only four EU member states will be able to include 95% zero-emission buses in their bus fleet [20]. In separate studies, Hojnik and Ruzzier [21] and then Cai and Li [22] have shown that the high demand for sustainable products creates the conditions for the development of innovative equipment. Alsabry et al. have stated that heating systems must increasingly consider the use of renewable energy sources. Heat pumps, together with solar panel installations, represent the most economically advantageous option [23]. Moreover, the same author argues that the transition towards the use of optimal sources of alternative energy requires significant capital and must be done according to economic, ecological, but also technical criteria [23].

The clean energy transition topic is also found in earlier research such as Verbruggen et al. [24]. Shifting from conventional energy sources to renewable energy stands as the primary hurdle in the initial decades of the 21st century. The energy transition encompasses three key aspects. The global energy system is currently experiencing a profound transformation in which three main objectives intersect: an economic objective involving the balance between supply and demand and the competitiveness among nations; a security objective driven by the strategic reliance on oil and gas trade; and a sustainability objective focused on achieving a low-carbon energy mix [25]. Popescu et al. concluded that the reduction of the carbon footprint forces the modernization of the industry, the increase of investments, especially those carried out through public procurement, but also the rethinking of business by merchants [26]. In a paper published in 2023, Rongrong Li et al. assert that "a negative relationship exists between renewable energy consumption and per capita ecological footprint" [27].

Gryzunova has suggested that the approach to investment projects in the field of green energy must strike a balance between consumer demand and the feasibility of providing specialized installations [28]. Through their study in China regarding green public procurement, Zhang and Jiang found that they stimulate companies to pay attention to the types of energy they use in the production process [29].

Azam et al. argued that meeting energy efficiency requirements entails high equipment and installation costs. Although renewable energy installations have a small share, in the next 10 years their importance will increase significantly. In many countries, the rules regarding air pollution have been tightened, normative acts have been issued regarding not only air quality, but also the carbon footprint [1]. According to Gryzunova, state governments establish development strategies in order to reduce costs, increase investments in the use of renewable energy sources. Russia's target scenario is for investments in this field to represent, in the period 2022–2030, 1% of GDP [28]. Lyu et al. asserted that France has proposed, by law, to be zero GHG by 2050, giving up electricity generated with gas and coal [30]. Nathaniel and Abdul have shown that in Southeast Asian countries, the environmental footprint has been reduced from 1990 to 2016 through the use of renewable energy [31].

According to Dyr, Misiurski and Ziolkowska, energy transition policies can create energy justice issues [19]. Hernández defines energy justice as being composed of the following: the right to sustainably obtained energy, the right to the best quality energy infrastructure, the right to reasonable energy prices and the right to have continuous access to energy services [32].

Grover and Daniels identified differential residential electricity tariffs in Wales and the United Kingdom [33]. In the UK, Walker and Day studied energy policy inequities [34]. Through their paper, Zhou and Noonan proposed, in the US, a framework for connecting clean energy policy instruments with energy justice concepts [35].

According to the European Commission, the REPowerEU plan can only be implemented by meeting the actions and targets of the RRM and the 'Ready for 55′ package, defined in line with the ambitious goal of the European Green Deal to achieve climate neutrality by 2050 [36]. In this context, coordination of actions not only between plans, but also between the ways of implementation in the Member States is essential for the structural transformation of the EU energy system.

On one hand, it is expected that electricity from fossil fuels will be needed to support the growing demand due to the new dynamics in Eastern Europe. Compared to previous scenarios, the trajectory of the transition to clean energy and how the EU will achieve its climate targets will change: coal capacities could be used for a longer period and European gas consumption will have to be reduced more rapidly, limiting its use as a transitional fuel [37].

On the other hand, it can be seen that measures are being taken to increase power production, in line with the climate and environmental targets set by the EU Member States, and this concern includes renewable power sources (wind, photovoltaic, hydro, nuclear, geothermal), biogas and hydrogen. Currently, the power mix in Romania consists of 8% nuclear energy, 12% renewable energy, 14% oil and oil products, 30% natural gas, and 36% coal and coal products [38].

It is important to note the energy mix refers to the combination of various primary energy sources used to meet a country's total energy consumption needs. This includes not only electricity generation but also other forms of energy consumption like heating, transportation, and industrial processes. The energy mix typically comprises fossil fuels (oil, coal, and natural gas), nuclear energy, and renewable energy sources (hydro, solar, wind, geothermal, and bioenergy). The energy mix is based on the official data provided by the NSI, datasets from IND118A - Electric energy production by type of energy plant [39].

The different sources used to produce electricity are the focus of the power mix, which is a subset of the energy mix. The energy mix consists of solid and fossil fuels (17%), natural gas (16%), renewables, biofuels, biomass (48%) and nuclear (19%) [38]. The proportion of electricity generated from different sources, such as coal, natural gas, nuclear, hydroelectric, wind, solar, and other renewables, is accounted for in the power mix.

A number of studies have addressed the topic of energy usage and efficiency across different sectors and geographies. These have been summarized in Table 1. The main findings indicate energy efficiency disparities among European countries. Also, there is a notable shift towards renewable energy sources and a decrease in fossil fuel use in the industrial sector.

**Table 1:** Research on forecasting and using energy from different sources and sectors in the EU.

Authors	Year	Methodology	Main Findings
J. Malinauskaite et al. [40]	2020	Review of EU's framework (Energy Efficiency Directive) and national plans, measures and policies in Slovenia and Spain.	Spain and Slovenia have great potential for industrial energy efficiency as they depend heavily on imported energy sources. These countries have set a more ambitious targets for 2023 comparing with the ones set for 2020.
J. Malinauskaite et al. [41]	2019	Analysis of EU and national policies from UK and Italy, regarding energy efficiency. Case studies on Italy and UK national policies.	Long-term legal predictability is required in energy efficiency policies. White Certificates play a major role in Italy's industrial energy efficiency. The UK's long-term policy targets decarbonizing the industry, with energy efficiency being a central element of a low-carbon economy.
P. Bertoldi and R. Mosconi [42]	2020	Econometric model to estimate energy savings, a dynamic panel model.	Energy policies seem to contribute to the reduction in energy consumption. EU energy consumption in 2013 would have been 12% higher without efficiency policies.
F. Al-Mansour [43]	2011	Analysis of energy consumption and efficiency indicators in Slovenia.	Improvements in energy efficiency indicators are due to policy measures. The government needs to prepare a new action plan with more innovative measures to achieve its obligation of energy efficiency improvement.
J. Brodny, M. Tutak [44]	2022	Statistical analysis using Eurostat data, descriptive statistics, Gini coefficients, Lorenz curves, Kohonen's neural networks	Significant disparities in energy efficiency and consumption patterns are found among EU states. Notable increase in energy consumption from renewable sources and decrease in fossil fuels use in the industrial sector are stated.

The transition in the energy sector to a new industrial phase requires an alignment of directives and targets, coupled with regulatory and monitoring measures, to harmonise the economic dynamics between producers and consumers. Renewable electricity generation capacity is a declared priority at the European level. For example, in the NRRP, Romania commits to phase out coal and lignite by 2032 and replace them with renewable and low-carbon energy sources.

The main challenges include the sustainable increase of green energy production capacity through the construction of new power plants, the integration of new forms of energy into the energy system, and the management of the variability of wind and solar energy sources, the most intensively used in the transition, which translates into the need to ensure the continuous supply of the electricity system. Strengthening electricity generation, distribution and supply systems that are ready to integrate renewables and green fuels as efficiently as possible requires significant investment both in the development of each Member State's energy system and in the adoption, integration and maintenance of advanced IT systems that ensure good generation management. Radulescu, Gâf-Deac and Bran argued that Romania needs to broaden its energy blend, enhance its domestic interconnection capability (which presently stands at less than 10% for electrical networks), and update its energy transmission grids [45].

The authors have also reviewed the previous research on the topic of energy forecasting. These have been summarized in Table 2.

**Table 2:** Research on energy forecasting.

Authors	Year	Methodology	Main Findings
Günay, M. E [46].	2016	Multiple linear regression and artificial neural networks (ANNs) based on variables like population, GDP per capita, inflation, unemployment, and average temperatures.	Unemployment percentage and average winter temperature were not significant predictors for 1975–2013. Future electricity demand is projected to double by 2028.
Yukseltan et al. [47]	2017	Linear regression model incorporating seasonal harmonics, without reliance on climatic or econometric data.	The model successfully predicts daily and weekly demand with about 3% error and provides insights into industrial vs domestic consumption.
Pérez-García & Moral-Carcedo [48]	2016	Index decomposition methodology to identify key factors influencing electricity demand and develop a long-term forecasting model.	Highlights the importance of considering various determinants of electricity demand rather than solely relying on GDP growth projections.
Al-Bajjali and Shamayleh [49]	2018	Vector Error Correction Model (VECM) analysing factors including GDP, electricity prices, population, urbanization, economic structure, and water consumption.	GDP, urbanization, economic structure, and water consumption have a significant positive impact on electricity consumption, while electricity prices have a negative effect.
Johannesen et al. [50]	2019	Comparative analysis of Random Forest Regressor, k-Nearest Neighbour Regressor, and Linear Regressor for short-term and long-term electrical load forecasting.	The Random Forest Regressor is more accurate for short-term predictions; k-Nearest Neighbour Regressor is better for long-term forecasting.
Sajid et al. [51]	2021	Regression Analysis and Monte Carlo Simulation	Authors have recommended energy policy guidelines for sustainable energy resource management in Pakistan.

The main findings indicate that there are a variety of statistical tools that the researchers have employed in order to forecast energy production or consumption, either in short term or in long term. Also, a significant number of variables have been evaluated for their effects on either production or consumption of electricity demand and supply.

## 3 Materials and methods

To provide answers to the above-mentioned research questions, the research methodology is based on a mixed approach, combining documentary analysis, literature review and predictive modelling, which is often used in energy studies. Creswell has argued that integrating both quantitative and qualitative research leads to a stronger understanding of the analysed problem [52]. A comprehensive analysis was conducted utilizing official datasets pertaining to electricity production in Romania, meticulously sourced from the National Institute of Statistics (NIS). These datasets served as the foundation for the development of a robust forecasting model targeting these critical indicators. As an initial step, a statistically grounded model was designed to predict Romania's electricity production values for the year 2023, drawing on historical data gleaned from the National Institute of Statistics' online TEMP database [39].

Moreover, this study meticulously examined the inherent uncertainty associated with estimating the 2023 electricity production levels. To achieve this, a Monte Carlo simulation approach was employed, which facilitated the generation of a probability distribution function graph.

The Monte Carlo simulation method has been used in different contexts to evaluate the annual energy production (AEP) of wind energy sources [[53], [54], [55], [56], [57], [58]].

This innovative simulation technique afforded the authors the capability to model and elucidate the multifaceted uncertainties and variabilities intrinsic to diverse energy sources. The dataset for this simulation encompassed production levels attributed to each distinct energy source, ranging from thermoelectric and hydroelectric to wind, solar, and nuclear electric. Input variables were thoughtfully selected to encompass the spectrum of nature, encompassing variables, negative and positive risks, the lowest estimate (LE), the most likely estimate (MLE), and the highest estimate (HE).

The simulation model is based on probabilities and random distributions in order to estimate the level of energy production in 2023. The model takes into account uncertainty factors such as negative and positive risks and the coefficient of variation. A simple random simulation method was used with 50,000 iterations to obtain the distribution of simulation results. This approach allows us to obtain sufficiently accurate results and ensure convergence.

The software used is Microsoft Excel with Visual Basic for Applications (VBA) to implement some macros and to perform the Monte Carlo simulation. The Monte Carlo simulation methodology, described in detail by Robert & Casella, was used to estimate energy production and consumption levels in future years [59] below. The model gives an estimate of the expected throughput of the energy mix [60] below.

The distribution used was a triangular one [61] below. The estimation of the random variate for the standard triangular x’ ∼ T (0, 1, $\tilde{x^{\prime }}$) is realised based on the following routine [60]:

1. Generate a random u ∼ U (0, 1).
2. If $u\leq {\tilde{x}}^{\prime },x^{\prime }=\sqrt{u{\tilde{x}}^{\prime }}$.
3. If $u>{\tilde{x}}^{\prime },x^{\prime }=1-\sqrt{\left(1-{\tilde{x}}^{\prime })(1-u\right)}$.
4. Return x’.

Simultaneously, the authors conducted a comprehensive analysis of the primary energy reports published by various pertinent international organizations operating at the European level. This document analysis, in line with the approach presented by Bowen, provided a broad perspective on the situation, complementing and enriching the analysis of statistical national data [62].

Ultimately, the authors employed a meticulously curated dataset acquired from the Electronic Public Procurement System (EPPS), encompassing information pertaining to public procurement activities undertaken in Romania during the period spanning from 2018 to 2022. This dataset facilitated the identification and examination of contracts specifically oriented toward the provision of alternative energy sources, allowing for a comprehensive analysis of their trajectory and evolution over time.

According to Law No. 98/2016 on Public Procurement [63], contracting authorities are required to conduct the procurement procedure for awarding contracts above certain values through EPPS, which can be accessed at www.e-licitatie.ro [64]. By querying the EPPS platform, section "award notices", by different search words ("energy", "geothermal water", "buses"), for the period 01.01.2018–31.12.2022, the system generated approximately 285 concluded contracts. For the analysis of these purchases, the authors of this article made the following generic groupings.

- by the subject of the contract, in the supply of: geothermal water, photovoltaic systems and buses;
- by the field of activity of the contracting authorities: territorial administrative units, comprising local and county councils, defence, culture/education, social services, other public entities;
- by bus categories: combustion engine; electric; hybrid.

Public procurement serves as a pivotal instrument for nations striving to effectuate energy transformation, specifically the transition towards alternative energy sources. The examination of the Electronic Public Procurement System (EPPS) dataset is of paramount significance in this context, as it enables an insightful analysis of how public procurement dynamics can aptly mirror the metamorphosis transpiring within the energy sector. As Romania maintains a steadfast commitment to reducing emissions and embracing sustainable energy solutions, these transformative tendencies manifest themselves through discernible shifts in public procurement trends.

The application of environmental policies is of significant importance in identifying solutions to minimise the environmental impact of procurement [65]. The inclination toward a cleaner and more sustainable energy portfolio should be distinctly observable in the escalation of public procurement contracts associated with renewable and alternative energy sources over recent years. Furthermore, scrutinizing public procurement activities related to transportation, particularly the acquisition of buses (be they combustion engine, electric, or hybrid), provides an additional lens through which to gauge energy sector transformation. Given the substantial role that buses play in public transportation, an increase in the allocation of contracts toward electric and hybrid buses, relative to standard buses, symbolizes a resolute dedication to curbing the carbon footprint associated with public transit. This commitment forms an integral component of the broader energy transformation initiative.

## 4 Results and discussions

### 4.1 Results

#### 4.1.1 The analysis of the dataset for the future energy production simulation

Our analytical framework commenced with a foundational dataset sourced from the National Institute of Statistics (NSI), encompassing annual electricity production statistics in Romania. This comprehensive dataset comprises various energy categories, including thermoelectric, hydroelectric, wind, solar, and nuclear electricity. To ensure the rigor of our analysis, each energy category underwent scrutiny over specific and relevant time intervals. To acquire this data, the authors methodically extracted information from the NSI's official website, focusing on the statistical indicator denoted as IND110A - Balance of electric energy by component elements. This indicator serves as a pivotal resource in the examination of electricity production dynamics in Romania.

**Fig. 1:**
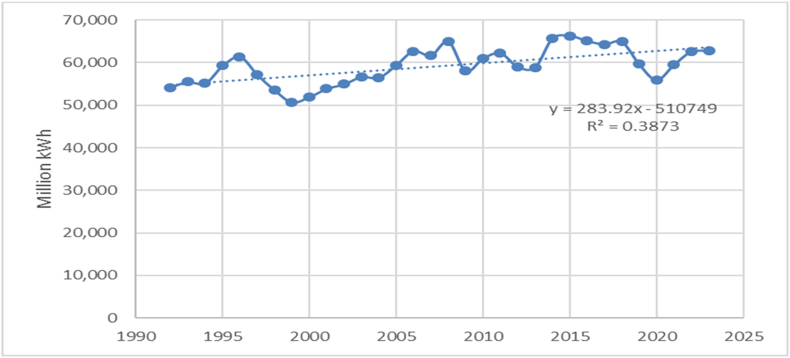
Total energy production for Romania between 1990 and 2021.

Source: Authors, based on NIS [39].

The production and consumption of electricity in Romania is on an increasing, stable trend, shown in Fig. 1. In this graph, the total energy production over an extended time span (1990–2021) is illustrated and several conclusions can be drawn. At first glance, there seems to be an increase in electricity production from the early 1990s to the mid-2000s, followed by a slight decrease and then an increase in the 2010s. Since 2015, there seems to be a general downward trend. From the lowest recorded production of 50.713 million kW/h in 1999 to the highest production of 66,296 million kWh in 2015, there is a significant variation in Romania's electricity production. There are also considerable fluctuations from year to year, indicating that energy production can be influenced by a number of dynamic factors. A significant increase in energy production occurred between 1994 (55,136 million kW/h) and 1996 (61,350 million kWh). In contrast, a sharp decrease occurred between 2008 (64,956 million kWh) and 2009 (58,016 million kWh).

Looking at the last decade of data (2011–2021), an overall decrease in energy production is observed. However, there were also periods of increase, such as 2014 (65,675 million kWh) compared to 2013 (58,888 million kWh). Fig. 1 also shows the regression equation y and the R^2^, which explains how well the regression model explains the actual observed data. In recent years, from 2020 to 2021, an increase in energy production has observed from 55,935 million kWh to 59,470 million kWh, which could indicate a reversal of the downward trend. This analysis does not consider the larger context of energy production in Romania that could include other influencing factors such as changes in national or international energy policies, the demand for energy, the development of underlying technologies or an economic outlook. In the following paragraphs, the electricity production from different sources will be discussed, based on the data extracted from the NSI website for the statistical indicator IND1180A – Electrical Energy Production by Power Plants.

**Fig. 2:**
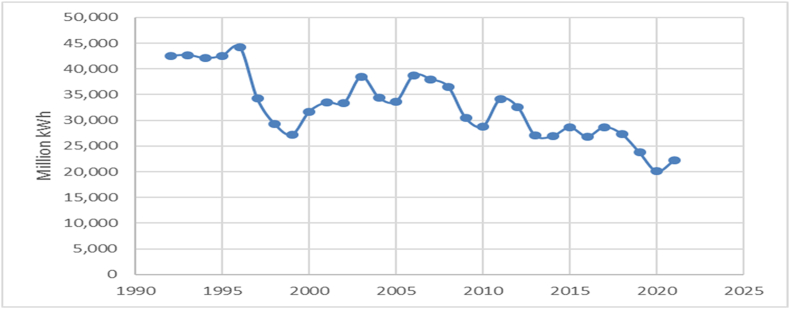
The electricity production by thermal power plants.

Source: Authors, based on NIS [39].

The evolution of the contribution of thermal power plants to electricity generation has been illustrated in Fig. 2. There seems to be a general downward trend in the energy production of thermal power plants in Romania from the early 1990s to the present. However, there are also periods of growth and stabilization. The variation in energy production is significant, with the lowest production recorded in 2020 (20,090 million kWh) and the highest in 1996 (44,209 million kWh). This indicates that the production of thermal power plants can be influenced by a number of dynamic factors.

A significant decrease in production occurred between 1996 (44,209 million kWh) and 1999 (27,225 million kWh). In contrast, a significant increase was observed between 1999 (27,225 million kWh) and 2003 (38,480 million kWh). Looking at the last decade of data (2011–2021), there is an overall downward trend in energy production, with some fluctuations. For example, production decreased from 34,136 million kWh in 2011 to 22,162 million kWh in 2021.

In recent years, from 2020 to 2021, there has been a slight increase in energy production from 20,090 million kWh to 22,162 million kWh. This could indicate a reversal of the downward trend, but will need to be confirmed with further data. The data seem to show a general downward trend in energy production from Termoelectrica from 1992 to 2021 (Fig. 2). However, there are significant fluctuations in this trend, with periods of increasing production.

The variability in production appears to be high, with a dramatic drop in production around 1997 and then again, an increase until 2006, followed by a general downward trend. Two significant decreases were observed between 1996 and 1997 and again between 2008 and 2009. In terms of increases, there was noticed a significant increase between 1999 and 2000 and again between 2005 and 2006. In the last decade (2011–2021), a general downward trend in production has been observed. However, there are fluctuations in this trend, with a temporary increase in production in 2017 followed by a continuous decrease until 2020. In more recent years (2019–2021), there has been a continued decrease in production from 23,786 million kWh in 2019 to 20,090 million kWh in 2020, followed by a slight increase to 22,162 million kWh in 2021.

**Fig. 3:**
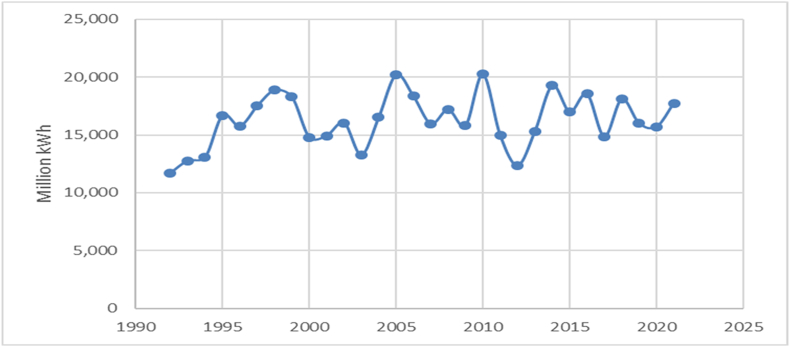
The energy production by hydroelectric power plants.

Source: Authors, based on NIS [39].

Fig. 3 shows the evolution of solar electricity production between 2012 and 2021 for Romania. It can be seen that after 2012, there is a strong upward trend until 2015, followed by a slight downward but relatively stable trend until 2021.

From 2012 to 2015, there was a considerable fluctuation in production, marked by a substantial surge. Post-2015, there is a minor fluctuation in production, characterized by a gradual yet relatively consistent decline. The most substantial rise is observed between 2012 and 2014, when production surged from 8 million kWh to 1616 million kWh. As for the decline, a gradual downward trajectory is noticeable post-2015, with production decreasing from 1982 million kWh in 2015 to 1703 million kWh in 2021. Over the past decade, solar energy production has experienced a remarkable growth, ascending from 8 million kWh in 2012 to a zenith of 1982 million kWh in 2015. Following this zenith, a gentle downward trend has ensued, yet production has largely maintained stability. In recent years, production has displayed relative steadiness, slightly diminishing from 1778 million kWh in 2019 to 1703 million kWh in 2021.

**Fig. 4:**
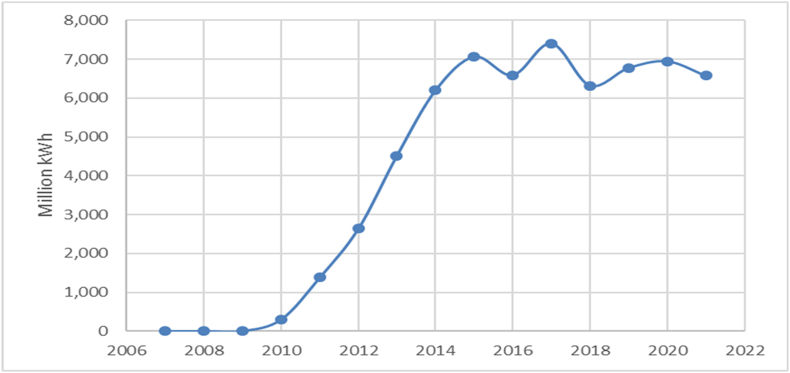
The energy production by the wind power plants.

Source: Authors, based on NIS [39].

Another substantial contributor to electricity generation in Romania is hydroelectric power plants. The evolution of this energy source is illustrated in Fig. 4.

Hydropower production has witnessed an ascent from 11,700 million kWh in 1992 to a zenith of 20,243 million kWh in 2010, characterized by notable fluctuations throughout the period. Post-2010, production appears to exhibit a general downward trajectory marked by fluctuations. Production displays year-to-year variability, featuring significant increments and decrements. This suggests that hydropower production might be subject to diverse influences, encompassing factors like rainfall patterns, shifts in energy demand, and alterations in energy policies. Substantial increases in production are noted between 1994 and 1995, followed by another surge between 2003 and 2005. The most substantial decrease is observed between 2010 and 2012.

**Fig. 5:**
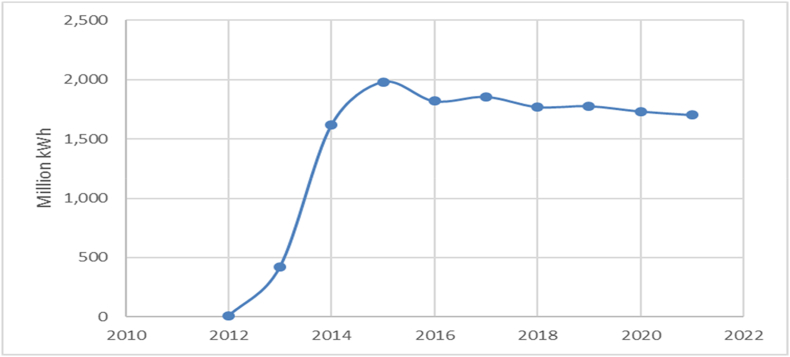
The solar energy production in Romania.

Source: Authors, based on NIS [39].

Throughout the past decade (2011–2021), the trend manifests fluctuations, experiencing a pronounced decline from 2010 to 2012, succeeded by alternating periods of production growth and reduction. Production in 2021 (17,745 million kWh) surpasses that of 2011 (14,946 million kWh), yet remains below the peak of 2010. In recent years (2019–2021), a slight upward trend in production becomes evident, ascending from 16,006 million kWh in 2019 to 17,745 million kWh in 2021.The last type of energy source that is analysed in this paper is the wind. The evolution of the energy production generated by the wind power plants is illustrated in Fig. 5.

The data show a general upward trend in wind energy production. Production started in 2007 at 3 million kWh and grew exponentially to 7063 million kWh by 2015. After 2015, the trend seems to be relatively stable with slight fluctuations. Production increased significantly between 2009 and 2013, with a growth rate that seems to have slowed down after 2015. The largest increase occurred between 2010 and 2013, with an increase from 306 million kWh to 4520 million kWh. After 2015, there is a minor variation, with a slight downward trend in 2016 and 2018. In the last decade (2011–2021), wind power generation has increased significantly from 1387 million kWh in 2011 to a peak of 7406 million kWh in 2017, with a stable trend since then. In recent years (2019–2021), wind energy production has been relatively stable, ranging from 6576 to 6945 million kWh.

To conclude this analysis, it can be noted that the transformation of Romania's energy sector has been marked by changes in energy production components. While traditional energy sources, such as thermal power plants, have shown a declining trend, renewable sources like solar and wind have been on the rise. This indicates a shift towards more sustainable energy options. The slight increase in hydroelectric power indicates the continued relevance of this energy source. Also, nuclear power has maintained a relatively constant share, at around 20% of total electricity generation. It's essential to recognize that the evolution of the Romanian energy sector results from an interaction of factors, including EU funding, legislation and incentives, foreign investments, geography, availability of natural resources, and environmental and climate change concerns. Each factor plays a varied role over time and across different energy sources.

### 4.2 The Monte Carlo simulation of energy production

In a Monte Carlo simulation, the true value of a variable is unknown and is described by a probability distribution. By running iterations of the simulation with randomly chosen values from this distribution, a distribution of possible outcomes can be obtained.

In Table 3, the authors have highlighted the Monte Carlo simulation model for energy production in Romania for the year 2023, by energy production components/sources. The columns "LE", "MLE", and "HE" represent the minimum (Lowest Estimate), most likely (Most Likely Estimate), and maximum (Highest Estimate) estimates of energy production for each source. The values for LE, MLE and HE form the basis of the probability model, helping the simulation of the process with the use of random values; for simplicity a triangular distribution has been used for the input. The "Negative" and "Positive" columns indicate potential negative and positive variations in energy production, likely based on certain risk or uncertainty scenarios. Finally, a total sum of the estimated energy production for all sources, both before and after the Monte Carlo simulation, is highlighted. This is a useful result as it gives an overview of the total energy production.

**Table 3:** The parameters of the Monte Carlo simulation.

Power Contributors	Total	Thermoelectric	Hydroelectric	Wind	Solar	Nuclear Electric
Energy Production (Millions KW/h)	62,765.08	23010.08	16,258.00	9769.82	2443.18	11,284.00
Negative	–	−15%	−15%	−15%	−15%	−10%
Positive	–	+20%	+20%	+20%	+20%	+20%
LE	53,914.52	19,558.57	13,819.30	8304.35	2076.70	10,155.60
MLE	62,765.08	23,101.08	16,258.00	9769.82	2443.18	11,284.00
HE	75,318.10	27,612.10	19,509.60	11,723.78	2931.82	13,540.80

**Fig. 6:**
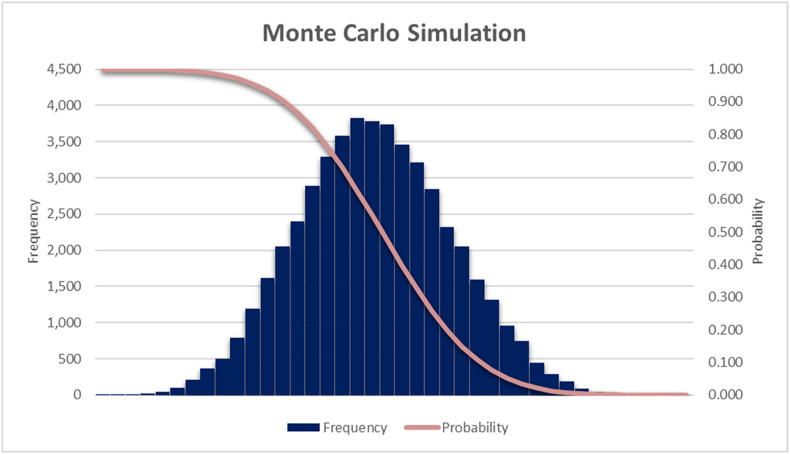
Monte Carlo simulation of the total energy production for 2023.

Source: Authors' modelling results

After 50,000 iterations, the results of the Monte Carlo Model for simulating the total energy production for 2023 are shown in Fig. 6.

The power production estimated with linear regression (62,765.08 million kWh) is higher than the production resulting from the Monte Carlo simulation with 90% probability (61,053.85 million kWh, as shown in Table 4). The simulation is based on the regression results and enhances accuracy, so it should be given the appropriate importance. The higher the projected production, the lower the probability of it being achieved.

**Table 4:** The projected power production and the associated probability.

Estimated Power Generation (Million kWh)	Probability (%)
56,182.82	99.96%
58,839.74	99.00%
60,168.21	95.00%
61,053.85	90.00%
64,153.59	50.00%

### 4.3 The analysis of the public acquisitions dataset

According to Regulation (EU) 2018/842, the problems generated by the energy crisis, the targets for the use of renewable energy sources and the targets for reducing greenhouse gas emissions oblige public institutions to identify and use alternative sources of electricity and heat, to reduce the consumption of fossil fuels, especially gas and oil [66]. The construction of wind farms and photovoltaic systems for electricity generation, the use of geothermal water for heating buildings and water, the purchase of electric cars and buses are ways of achieving the objectives of replacing traditional sources of energy and fuels. The state, through its public institutions, being a major energy consumer, is increasingly concerned about acquiring alternative energy sources.

High electricity costs have led contracting authorities to use alternative sources of electricity, such as solar and geothermal energy. Thus, since 2021, authorities have increasingly turned to the use of solar energy, purchasing photovoltaic panel systems and installing them on the roofs of public institutions and households. Photovoltaic systems have also been a way of electrifying homes in hard-to-reach areas. Funding for the program "Increasing Energy Efficiency of Buildings" has contributed to the purchase of photovoltaic power generation installations.

However, in the period 2018–2022, the number of public procurement contracts, which were for the supply of alternative energy sources, was reduced, with contracts for the supply of photovoltaic systems in EPPS amounting to 35,346,819 RON and contracts for the purchase of geothermal water amounting to 7,695,562 RON (Table 5). The data in the table relate to the contract values at the time of award, without inflation adjustment.

**Table 5:** The value of the contracts related to the alternative energy sources (RON).

Energy source	2018	2019	2020	2021	2022	Total
Geothermal Water	734,782	305,306	2,742,403	551,263	3,361,809	7,695,562
Solar Energy	63,910	302,856	2,626,178	11,843,727	20,510,148	35,346,819
Total	798,692	608,162	5,368,581	12,394,990	23,871,957	43,042,381

Although there are significant hot springs underground in Romania, geothermal energy is not used enough to heat buildings and water. Through EPPS, contracts for geothermal water supply have been concluded over the last 5 years, mainly by military units and public tourism and leisure services. The authors believe that there is great potential for using this energy source in the Romanian energy mix.

The crisis generated by COVID 19, which also impacted fuel shipments, and the energy crisis caused by the war in Ukraine, have led to an accelerated increase in solar plant purchases in 2021-2020. If in 2020, in EPPS, contracts worth 2,626,178 RON are awarded, in 2021 their value is 4.5 times higher and in 2022 about eight times higher. Most contracts were concluded by the territorial administrative units, mostly in rural areas. Also, various categories of public institutions (education, social services, universities, autonomous regions, etc.) were interested in installing solar panels on buildings (Table 6).

**Table 6:** Beneficiaries of alternative energy sources (RON).

CA field	2018	2019	2020	2021	2022	Total
ATU			2,096,798	11,580,284	8,592,563	22,269,645
Public enterprises	63,910	608,162	551798.61	685,711	11,256,828	13,166,410
Defence	734,782		2,190,604		2,764,178	5,689,564
Culture/education				128,995	1,195,896	1,324,891
Other			529,380			529,380
Social services					62,491	62,491
Total	798,692	608,162	5,368,581	12,394,990	23,871,956	43,042,381

Examining the annual total, there is a general increase in the contractual values of beneficiaries from the use of alternative energy sources, increasing from 798,692 RON in 2018 to 23,871,956 RON in 2022. This shows an increased commitment from Romania to rely more on alternative energy sources. It can be noted that the fields "Culture/Education" and "Social Services" started to benefit from alternative energy sources only recently, in 2021 and 2022, respectively. This suggests that the implementation of alternative energy sources in these fields could be at an early stage, or that they had other funding priorities until recently.

In general, from the above, it can be seen that there is a commitment in Romania to the use of alternative energy sources, but the pace and level at which each institution adopts these sources vary. Given the importance of the transition to alternative energy sources for achieving climate and energy goals, it is desirable that this commitment continues and expands across all public sectors.

One cause of rising carbon emissions is the use of fossil fuels in transport. According to European Environment Agency, between 1990 and 2019, transport emissions increased by more than 33% due to transport [67]. Switching to electric cars is one way to reduce pollution. In Romania, in the period 2018–2022, in EPPS contracting authorities registered contracts for the purchase of buses (including electric charging stations) in the amount of RON 3330 million (Table 7).

**Table 7:** The value of the buses purchased expressed (RON).

Object	2018	2019	2020	2021	2022	Total
Buses	40,761,529	64,532,127	597,694,868	57,126,216	261,308,502	1,021,423,241
Electric buses	4,700,000	33,806,000	216,755,492	706,523,910	766,885,884	1,728,671,286
Hybrid buses		211,380,074	189,118,142	89,450,250	59,747,818	549,696,284
Electric charging station	2,104,570	4,038,469	3,973,666	8,508,731	11,603,759	30,229,195
Total	47,566,099	313,756,669	1,007,542,168	861,609,107	1,099,545,963	3,330,020,007

The data presented in Table 7 illustrates the progression of public contract values for the acquisition of buses and related infrastructure during the period of 2018–2022. The data highlights a noteworthy surge in investments directed towards buses, particularly those of the electric and hybrid variants. In 2018, the investments primarily concentrated on conventional buses, amounting to 40,761,529 RON. Starting in 2019, a consistent uptick in investments towards electric and hybrid buses becomes evident, culminating in peak values for 2020 and 2021, respectively. This trend underscores an amplified dedication to adopting more environmentally friendly modes of transportation, reflecting the inclination of Romanian public authorities to emphasize the utilization of renewable energy sources.

**Fig. 7:**
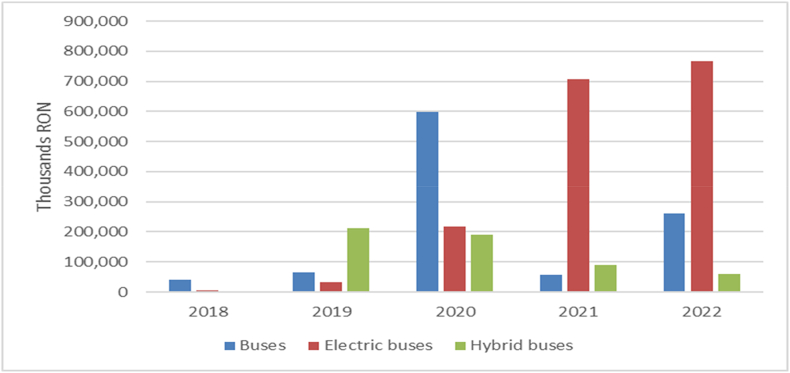
The value of the buses purchased.

Source: Authors, by using EPPS

Investments in electric charging stations have likewise experienced an upsurge, reaching its peak value in 2022, underscoring the necessity for appropriate infrastructure to support electric buses. This trend highlights the ongoing transformation of the public transport sector toward more sustainable and ecologically conscious solutions, with a pronounced shift toward electric and hybrid buses. Starting in 2021, contracting authorities have shown a preference for procuring electric and hybrid buses over traditional fuel-powered ones. Consequently, fuel-powered buses are being gradually replaced by electric and hybrid counterparts, as depicted in Fig. 7.

These patterns within public procurement contracts vividly mirror Romania's steadfast commitment to overhauling its energy sector, particularly by mitigating carbon emissions within public transportation. The principal driver behind the surge in energy-related purchases lies in the allocation of non-repayable funds through diverse programs designed to achieve sustainable development objectives.

Notably, the increase in fuel and electricity prices in 2022, attributed to the conflict in Ukraine, also played a substantial role, prompting a shift towards the procurement of electric or hybrid buses. These trajectories within public procurement agreements distinctly mirror Romania's resolute dedication to reshaping its energy landscape, specifically by curtailing carbon emissions stemming from public transportation.

The energy sector in Romania will receive funds via the National Recovery and Resilience Plan (NRRP). These funds are as high as €855 million and should be spent on investments in clean energy production and reducing the dependence of power production on coal and lignite. In addition to this, significant amounts are allocated for sustainable transport, including the purchase of electric/hydrogen buses and recharging stations.

As shown previously, the energy sector in Romania will receive significant funds to transition to clean energy via the National Recovery and Resilience Plan (NRRP). The plan involves spending €855 million on reforms and investments to promote clean energy production, including phasing out coal and lignite power production and investing in renewable energy and hydrogen [7]. An additional €2.7 billion is allocated for enhancing the energy efficiency of buildings [7]. These funds will significantly impact the energy sector, supporting the transition to renewable energy sources and improving energy efficiency.

The Romanian authorities are actively planning to switch to clean energy production. This has also been demonstrated by growing investments and the procurement of electric and hybrid buses. There has been a notable growth in investments towards these environmentally friendly modes of transportation from 2019 onwards.

There has also been a general increase in the contractual values of beneficiaries from the use of alternative energy sources, showing a growing reliance on these sources across various public sectors. However, the pace at which each institution adopts renewable energy sources varies, suggesting a gradual transition.

## 5 Discussions

The current research has used Monte Carlo simulation in the energy sector, like some of the previous research [[53], [54], [55], [56], [57], [58]]. However, these had different applications (e.g., wind farms or wind power plants [[55], [56], [57]]) and did not include all the energy production components at the national level [58]. Furthermore, even if the regression analysis has been combined with the Monte Carlo simulation method [51], this does not apply to the Romanian energy landscape.

This analysis of public procurement data within Romania's energy sector accentuates its pivotal role in driving these transformations. The contours of public procurement patterns furnish an insightful vantage point for policy formulation, investment strategizing, and the monitoring of progress, functioning as a reflection of the nation's energy transition. The trends in public acquisitions suggest an expanding dedication in Romania towards the adoption of alternative energy sources. However, the rate at which each institution embraces these sources does vary. Given the imperative nature of transitioning to alternative energy sources to meet climate and energy objectives, it remains crucial for this commitment to be both sustained and widespread across all public sectors.

Romania stands to gain significant advantages by further investing in the expansion of its green energy production capacity, curbing sources of pollution, and bolstering building energy efficiency in a sustainable manner. These investments, frequently facilitated through public procurement, carry substantial weight in bolstering national economic growth and fostering job opportunities. The shift from conventional energy to sustainable alternatives presents an avenue for the evolution of the energy sector. Through public procurement agreements concerning the provision of geothermal water, awarded over the past five years, Romania has effectively demonstrated its considerable potential for incorporating this energy source into the country's broader energy portfolio. This suggests that public procurement in Romania is a driver of the transition towards clean energy and environmentally friendly modes of transportation.

Furthermore, the study reveals a clear and growing commitment in Romania towards the adoption of alternative energy sources. The Monte Carlo simulation model projects 2023 energy production, giving a comprehensive outlook of energy production from all sources. Linear regression estimates suggest a potential for 62,765.08 million kWh production, compared to the Monte Carlo simulation, which indicates a 90% probability of achieving 61,053.85 million kWh. As projected production levels increase, the probability of achieving the production goal correspondingly decreases.

This commitment is evident in the trends observed in public procurement, highlighting the pivotal role of such initiatives in propelling Romania's energy transition. Despite the inherent uncertainties accompanying energy transformation, the study presents positive insights into Romania's potential for diverse sources of electricity production. In summary, public procurement emerges as a crucial instrument guiding the energy sector toward a greener and more sustainable future while simultaneously furnishing a valuable perspective for policy formulation, investment strategizing, and progress assessment throughout this critical phase of transition.

The unique perspective of the current paper is the inclusion of the public procurement element into the analysis, a research perspective previously unexplored in the Romanian energy sector. This contributes to the body of knowledge regarding the Romanian energy sector.

The current research facilitates the development of future research that includes a longer forecasting horizon, for example, until 2030 or beyond. Given the multiple variables of total annual energy production, the longer the forecasting horizon, the higher the chances are that the forecast will be inaccurate. Therefore, in addition to regression analysis and the Monte Carlo simulation method, other data analysis techniques may also prove their utility.

## 6 Conclusions

The European Commission has advised Romania to decrease its reliance on fossil fuels, a recommendation that aligns with the worldwide imperative for sustainable energy generation. This advice underscores the need for hastened progress in the advancement of renewable energy sources, the enhancement of energy transmission networks, and the enlargement of interconnections with neighbouring EU Member States.

The present study adds to a more comprehensive analysis of Romania's transition towards green energy by offering an overview and a potential projection of electricity production levels in 2023, accounting for inherent uncertainties.

The paper also places emphasis on the examination of public activities within Romania, executed via the EPPS public platform, considering that contract award regulations are standardized across all EU member states, originating from Directives No. 24/2014 and No. 25/2014. The study's findings underscore Romania's active involvement in fulfilling the sustainable development goals delineated in the National Recovery and Resilience Plan (NRRP). The realm of public procurement within the energy sector displays a consistent upward trajectory, as evidenced by the increasing prevalence of electric and hybrid buses, along with solar panels. This trend signifies Romania's commitment to sustainable development objectives. Outcomes derived from the analysis of EPPS data indicate that Romania is making noteworthy investments in energy transformation, presenting a potential opportunity for operators from various countries.

The study included a detailed analysis of energy production in Romania over the past decades. Graphical visualizations of the historical trends in energy production are essential for understanding the current and future state of the Romanian energy sector. In recent years, an increase has been noticed in annual energy production, from 55,935 million kWh in 2020 to 59,470 million kWh in 2021.

Another important point is the decrease in thermal power production. In the last decade (2011–2021), a general downward trend in thermal power production was observed. However, there are fluctuations in this trend, with a temporary increase in production in 2017 followed by a continuous decrease until 2020.

Furthermore, there is a rise in renewable energy sources with a variable evolution of hydroelectric and wind energy production. The study reveals significant fluctuations in these sources, indicating the variable nature of these renewable sources and their impact on overall energy production.

In conclusion, Romania's notable capacity for electricity generation from a variety of sources holds promising potential in the context of the energy transition. Nonetheless, to realize this potential, substantial investments in equipment, infrastructure, and transportation are imperative.

The research holds relevance for both commercial enterprises, aiding business development, and for decision-makers within Romania, expediting the energy transformation process. This process, carried out through public initiatives, can enhance living standards and generate new employment opportunities. Furthermore, the state's initiation of numerous public procedures acts as an engine for the advancement of diverse industry sectors, including the production of solar panels, electric and hybrid vehicles, buses, and more.

The main research limitation is that the analysis presented in this paper is confined to the case of Romania and focuses on specific aspects of the national energy regulations and procedures. The NRRP encompasses a diverse range of measures and objectives. In this analysis, our focus is directed towards the specific aspects related to the production of electricity from renewable sources, the beneficiaries of these alternative energy sources by the procurement field they belong to, and the investments made in green transportation.

For future research directions, it would be beneficial to conduct similar studies on other European countries in order to gain insights into the relative progress that the state members are making toward meeting the energy goals. Furthermore, the procurement contracts offer objective data concerning the specific actions undertaken by the member states during this period of transition. The availability of such studies that are focused on assessing progress can serve as a valuable source of information and inspiration for implementing more effective future measures in a space of shared knowledge among member states.

## Data availability statement

The datasets analysed during the current study are publicly available at http://statistici.insse.ro:8077/tempo-online/#/pages/tables/insse-table. Also, this research is based on publicly available data collected from Romania's Electronic Public Procurement System (EPPS). The datasets include detailed information on energy production and public procurement activities in Romania. Due to confidentiality agreements and data protection regulations, some of the raw data used in this study is not publicly accessible. However, in accordance with moral standards and applicable laws, the authors are willing to provide aggregated data and analysis results upon request.

The software used in Monte Carlo simulation is Microsoft Excel, with Visual Basic for Applications (VBA) that was used in order to implement macros necessary for automating the simulation. The excel file used for Monte Carlo simulation is available on Google drive: https://drive.google.com/file/d/1RVyt7q4JZrzbFbiNf8S2iKcHhPBJnpL8/view?usp=sharing.

## CRediT authorship contribution statement

**Mihai Ciobotea:** Writing – original draft, Software, Investigation, Formal analysis, Data curation, Conceptualization. **Ecaterina-Milica Dobrotă:** Writing – original draft, Resources, Investigation, Conceptualization. **Marian Stan:** Writing – original draft, Visualization, Validation, Methodology, Investigation, Formal analysis, Data curation, Conceptualization. **Delia Bălăcian:** Writing – original draft, Resources, Investigation, Conceptualization. **Silvius Stanciu:** Writing – original draft, Investigation, Funding acquisition, Conceptualization. **Adriana Dima:** Writing – review & editing, Writing – original draft, Investigation, Conceptualization.

## Declaration of competing interest

The authors declare that they have no known competing financial interests or personal relationships that could have appeared to influence the work reported in this paper.
